# Characterization of Asparagine Deamidation in Immunodominant Myelin Oligodendrocyte Glycoprotein Peptide Potential Immunotherapy for the Treatment of Multiple Sclerosis

**DOI:** 10.3390/ijms21207566

**Published:** 2020-10-13

**Authors:** Maria-Eleni Androutsou, Agathi Nteli, Areti Gkika, Maria Avloniti, Anastasia Dagkonaki, Lesley Probert, Theodore Tselios, Simona Golič Grdadolnik

**Affiliations:** 1 Vianex S.A., Tatoiou Str., 18th km Athens-Lamia National Road, 14671 Athens, Greece; androutsoum@vianex.gr; 2 Department of Chemistry, University of Patras, 26504 Patras, Greece; anteli@upatras.gr (A.N.); areti220@gmail.com (A.G.); 3 Laboratory of Molecular Genetics, Hellenic Pasteur Institute, 127 Vasilissis Sophias Ave., 11521 Athens, Greece; m.avloniti.17.5@gmail.com (M.A.); anastaciadk16@gmail.com (A.D.); lesley.probert@gmail.com (L.P.); 4 Laboratory for Molecular Structural Dynamics, National Institute of Chemistry, Hajdrihova 19, 1001 Ljubljana, Slovenia

**Keywords:** asparagine deamidation, nuclear magnetic resonance, myelin oligodentrocyte glycoprotein, experimental autoimmune encephalomyelitis, multiple sclerosis

## Abstract

Mannan (polysaccharide) conjugated with a myelin oligodendrocyte glycoprotein (MOG) peptide, namely (KG)_5_MOG_35–55_, represents a potent and promising new approach for the immunotherapy of Multiple Sclerosis (MS). The MOG_35–55_ epitope conjugated with the oxidized form of mannan (poly-mannose) via a (KG)_5_ linker was found to inhibit the symptoms of MOG_35–55_-induced experimental autoimmune encephalomyelitis (EAE) in mice using prophylactic and therapeutic vaccinated protocols. Deamidation is a common modification in peptide and protein sequences, especially for Gln and Asn residues. In this study, the structural solution motif of deaminated peptides and their functional effects in an animal model for MS were explored. Several peptides based on the MOG_35–55_ epitope have been synthesized in which the Asn^53^ was replaced with Ala, Asp, or isoAsp. Our results demonstrate that the synthesized MOG peptides were formed to the deaminated products in basic conditions, and the Asn^53^ was mainly modified to Asp. Moreover, both peptides (wild type and deaminated derivative) conjugated with mannan (from *Saccharomyces cerevisiae*) independently inhibited the development of neurological symptoms and inflammatory demyelinating spinal cord lesions in MOG_35–55_-induced EAE. To conclude, mannan conjugated with a deamidated product did not affect the efficacy of the parent peptide.

## 1. Introduction

Peptides and proteins play an important role in the field of medicinal chemistry and biomedical research. In the pharmaceutical industry, peptides are emerging as a novel class of therapeutic agents. To date, about 100 peptides are on the market in the USA, Europe, and Japan, as well as for diagnostics applications [1,2,3]. Peptides demonstrate significant advantages as potential pharmaceuticals over small molecules due to their high biological activity and high receptor specificity [4].

During the design and synthesis of peptides, one has to consider numerous interactions and behaviors of the constituting amino acids. A search of the literature revealed that in many cases, several types of impurities might be encountered in synthetic peptides. Impurities can either originate from raw materials during the manufacturing process or be formed during storage [5]. In many cases, during the characterization of synthetic peptides, detectable byproducts lack one or more amino acid residues in the desired peptide sequence. It was noticed that Met, Trp, and Cys amino acids are sensitive to oxidation. The oxidation of a Cys-containing peptide yields a disulfide bridge to form cystine; Met is converted into a sulfoxide [6]. Both transformations are reversible. Tyr and Trp in peptide sequences should be protected from sunlight, as both amino acids are at risk of photo-oxidation [7]. The rate of racemization in solid-phase peptide synthesis is common and depends on many factors, including the electron-withdrawing effect of the amino acid side chain, the temperature, the reaction solvent, and the coupling reagents [8,9,10,11]. Acetylation of amino functions could occur in acidic conditions with the treatment of trifluoroacetic acid. This reaction is reversible in mild alkaline conditions [12]. Other side-reactions in peptide synthesis address the Trp reduction by trialkyl silane and the irreversible alkylation on the Cys side chain functional group during the deprotection step [13].

Deamidation of Gln and Asn presents a frequent modification in peptide and protein sequences. This reaction is favored in neutral and mostly basic solutions. Deamidation of Asn leads to a mixture of L, D-succinimidyl, L, D-aspartyl, and L, D-iso aspartyl with the greater presence of L-iso aspartyl [14]. The new product adds to the mass of intact molecule + 0.984 Da the difference between the mass of hydroxyl (-OH) and amine (-NH_2_) functional groups. The intermediate cyclic imide succinimide appears with a different molecular weight, ~17–18 Da less [15,16].

Slight modifications in the sequence can result in drastic changes in the structure and function of peptides. For example, a modified residue such as deaminated Asn could positively or negatively affect the peptide/protein potency, efficacy, and safety [17,18,19,20,21]. Thus, deamidation should be monitored using several analytical techniques such as LC-MS/MS analysis, capillary electrophoresis, and NMR [22].

The presented study involves the investigation of deaminated products in combination with experimental autoimmune encephalomyelitis (EAE, animal model of multiple sclerosis (MS)), the evaluation of 35–55 myelin oligodendrocyte glycoprotein (MOG_35–55_) epitope that contains the -Asn^53^-Gly^54^- sequence. MOG is a glycoprotein located on the external surface of the myelin sheath [23,24,25]. The 35–55 epitope of rat MOG (MOG_35–55_: H-Met^35^-Glu-Val-Gly-Trp-Tyr-Arg-Ser-Pro-Phe-Ser-Arg-Val-Val-His-Leu-Tyr-Arg-Asn^53^-Gly-Lys^55^-OH) induces chronic EAE in mice of the C57BL/6 strain and is widely used for in vivo biological evaluation and immunological studies relevant to MS treatment [26,27,28,29]. The immunogenic MOG_35–55_ epitope conjugated to polysaccharide (mannan) via the (KG)_5_ bridge at *N* terminal protected mice against EAE when administered in prophylactic and therapeutic protocols [30,31,32,33,34,35,36,37]. Combinations of different peptides from myelin proteins (including MOG peptides) have been used in clinical studies against MS [38]. Moreover, MOG_35–55_ is one of the immunodominant epitopes that, conjugated to peripheral blood monocytes, resulted in a decrease of antigen-specific T cell responses in MS patients [38]. The structure of peptides based on the Asn^53^ transformation of synthesized MOG_35–55_ peptides has been explored. Model peptides with mutations of Asn with Ala, Asp, or isoAsp were synthesized and analyzed with 2D NMR spectroscopy in neutral and basic aqueous solutions. The comparison of chemical shifts and NMR signal patterns reveals the Asn^53^ deamidation to Asp in basic conditions while it is not revealed in neutral solution. Furthermore, the modified peptide containing Asp at position 53 showed similar biological activity compared to that of the wild type peptide against EAE when the peptides are conjugated to mannan polysaccharide.

## 2. Results and Discussion

### 2.1. Design and Synthesis of Peptides Based on the Immunodominant MOG_35–55_ Epitope

MOG_35–55_ is the main immunodominant epitope of MOG protein and is of great interest in our research regarding the peptide-based EAE treatment. This epitope conjugated with the oxidized form of mannan (poly-mannose) via a (KG)_5_ linker was found to inhibit the EAE symptoms in mice using prophylactic and therapeutic vaccinated protocols [30,31,32,33,34]. The conjugation reaction of (KG)_5_MOG_35–55_ peptide analog with oxidized mannan was performed in bicarbonate buffer solution, pH 9.0. In the basic conditions of conjugation reaction, the deamidation of Asn^53^ may occur. Model peptides were designed and synthesized (Pep 1–Pep 4), as shown in Table 1, to investigate the possibility of the formation of deaminated products during conjugation reaction. The obtained results lead to further investigation, and the (KG)_5_MOG_35–55_ peptides with Asn or Asp or isoAsp, respectively (Pep 5–Pep 7; human sequence) at position 53 were synthesized. A combination of reverse-phase high-performance liquid chromatography (RP-HPLC) and NMR were used to confirm the existence of deaminated products. Furthermore, it was interesting to examine how the chemically modified peptides would affect the immunological properties of the native peptide. Therefore, the rat sequence of (KG)_5_MOG_35–55_ (Ser^42^) (Pep 8) and the deaminated derivative containing Asp at position 53 (KG)_5_MOG_35–55_ (Ser^42^, Asp^53^) (Pep 9) were synthesized and conjugated with the oxidized form of mannan (Con-Pep 8 and Con-Pep 9). Both conjugates were evaluated in vivo in C57BL/6 mice using the EAE animal model of MS.

**Scheme 1:**
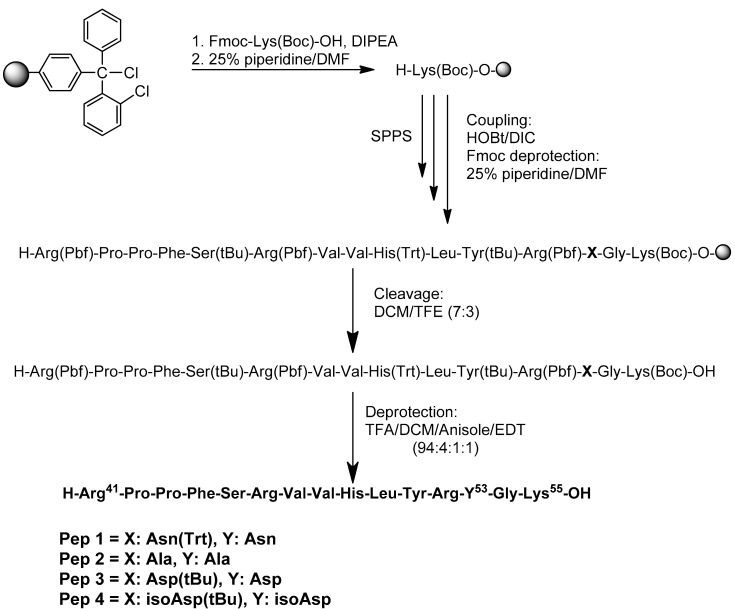
Representative SPPS of model peptides (Pep 1–Pep 4). The same synthetic procedure was followed for the synthesis of Pep 5–Pep 9 peptides.

The Pep 1–Pep 9 peptides (Table 1) were synthesized by solid-phase peptide synthesis (SPPS) in combination with the Fmoc/tBu methodology and using acid-labile 2-chlorotrityl chloride (CLTR-Cl) resin and *N^α^*-Fmoc (9-fluorenylmethyloxycarboxyl)-protected amino acids, as previously described and presented in Scheme 1 [39,40,41,42,43,44,45,46]. The first *N^α^*-Fmoc protected amino acid was esterified to the CLTR-Cl resin in the presence of diisopropylethylamine (DIPEA) and followed by Fmoc deprotection. The peptide bonds were created using 1-Hydroxybenzotriazole (HOBt) and *N,N′*-Diisopropylcarbodiimide (DIC) as coupling reagents [39,40,41,42,43,44,45,46]. The final deprotection of each peptide was achieved using trifluoroacetic acid (TFA) in dichloromethane (DCM) in the presence of scavengers (Scheme 1). The synthesized peptides were further purified and identified using semi-preparative RP-HPLC and electron spray ionization mass spectrometry (ESI-MS), respectively. The purity of peptides was more than 95%, as determined by analytical RP-HPLC.

### 2.2. HPLC Analysis

**Figure 1:**
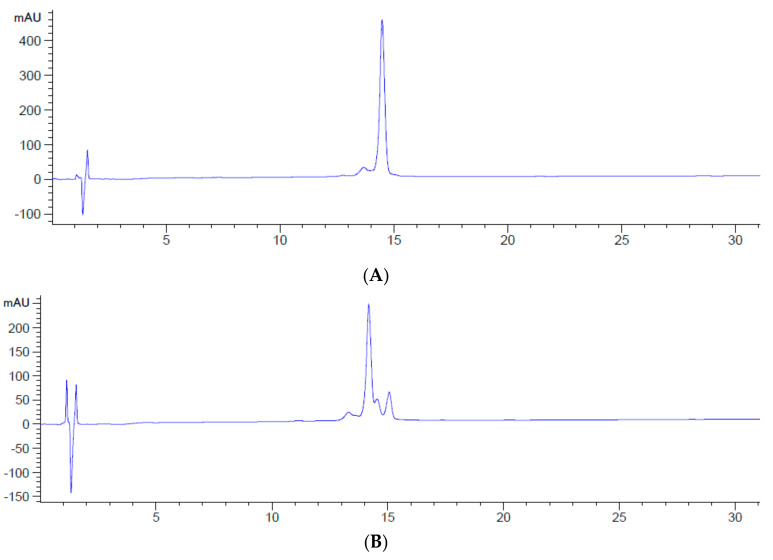
RP-HPLC chromatogram of (KG)_5_MOG_35–55_ (Ser^42^) (Pep 8) at 214.4 nm and 48 h after dilution; (**A**) dissolved in water, (**B**) dissolved in bicarbonate buffer pH 9.0. The deaminated product was revealed as a new peak to the right of the main peak.

The HPLC analysis of synthesized peptides was achieved using the analytical HPLC system (1260 Infinity, Quaternary Pump VL Agilent, Waldbronn, Germany) equipped with the Purospher RP-18 column (5 μm, Hibar 100 × 4.6 mm, Darmstadt, Germany), followed by UV detection at 214.4 nm and 254 nm. Separation was achieved by gradient elution from 18% acetonitrile (AcN) to 40% AcN over 30 min at a flow rate of 1 mL/min. The analysis demonstrated that the peptide with Asn residue at position 53 has been degraded and provided two new peaks at the right of the main peak in HPLC chromatogram after dilution in bicarbonate solution pH 9.0 (Figure 1 and Figure S1). The relative retention time of the (KG)_5_MOG_35–55_ (Ser^42^) deaminated analog, based on HPLC chromatogram (Figure 1), is 1.06, and this was further investigated during the present study.

The possibility of deamidation of Asn was apparent since the literature has addressed studies related to this formation [14,15,16,17,18,19,20,21,22]. Several peptides were synthesized based on 35–55 epitope of MOG (Pep 1, Pep 5, and Pep 8); with substitution of Asn residue with Ala (Pep 2), Asp (Pep 3, Pep 6, and Pep 9), and isoAsp (Pep 4, Pep 7), and they were analyzed by HPLC and ESI-MS (Figure 1 and Figures S1–S15). It was observed that the byproduct is revealed only in Pep 1, Pep 5, and Pep 8 peptides, containing Asn^53^, in basic conditions (carbonate buffer pH 9.0), and it was not detected in the other synthesized peptides with Ala, Asp, or isoAsp substitutions at the same position (Figures S1–S15). The deamidation formation is initiated upon dilution of the peptides in bicarbonate buffer solution and stabilizes after one day. The identified retention times in HPLC chromatograms of produced byproducts in basic conditions of peptides Pep 1, Pep 5, and Pep 8 matched the retention times of the synthesized peptides Pep 3, Pep 6, and Pep 9. This observation supports that Pep 1, Pep 5, and Pep 8 containing the Asn residue at position 53 undergo deamidation and semi-covert to the peptides Pep 3, Pep 6, and Pep 9.

### 2.3. NMR Analysis

**Figure 2:**
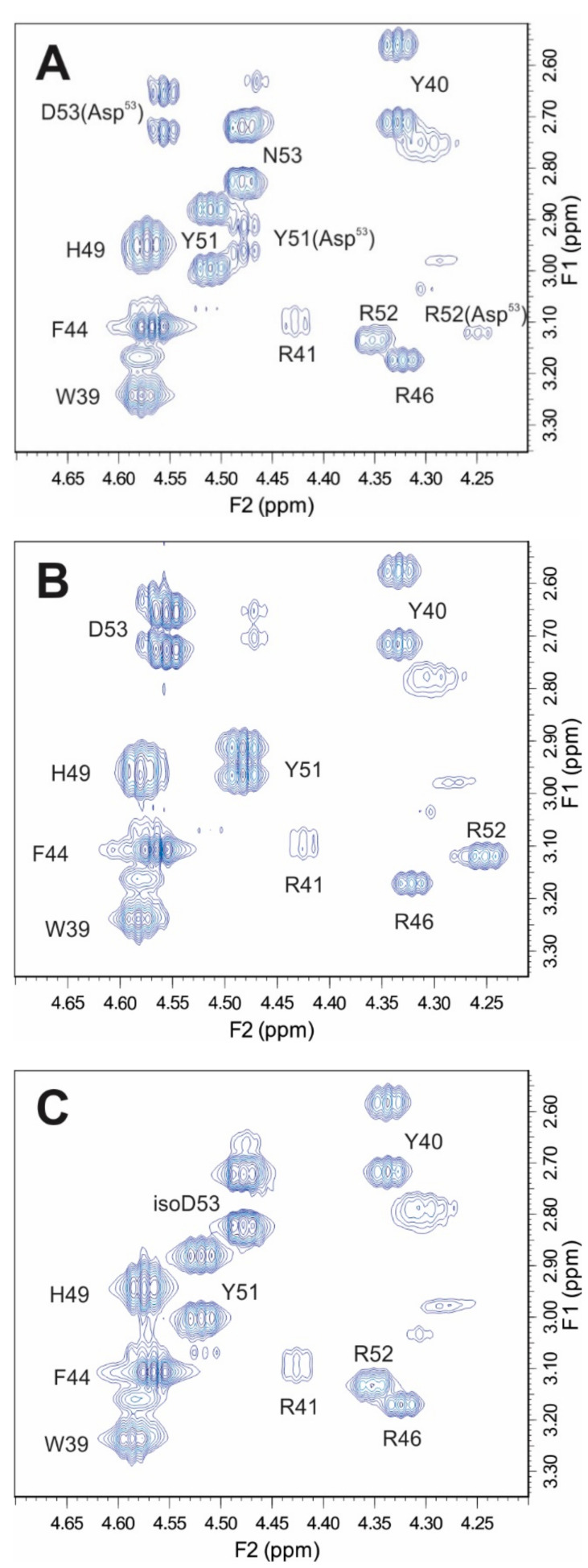
Expanded regions of Total Correlated Spectroscopy (TOCSY) spectra of: (**A**) (KG)_5_MOG_35–55_ (Pep 5), (**B**) (KG)_5_MOG_35–55_(Asp^53^) (Pep 6) and (**C**) (KG)_5_MOG_35–55_(isoAsp^53^) (Pep 7) recorded in bicarbonate buffer, pH 9.0.

The NMR chemical shift analysis of peptides 1–7 was performed to elucidate the nature of modification observed at HPLC analysis in basic conditions for peptides containing Asn at position 53. As the NMR chemical shifts are very sensitive to changes in the local chemical environment, the modifications related to Asn^53^ could also result in chemical shifts of neighboring residues. Indeed, expansions of the Total Correlated Spectroscopy (TOCSY) spectra recorded in bicarbonate buffer pH 9.0, which contain well separated ^1^H α-β cross-peaks of residues 51 and 53 and α-δ cross peaks of residue 52 of all investigated peptides, came out as the most demonstrative to elucidate the modification process (Figure 2). The positions and appearance of these cross-peaks critically depend on the modification of Asn^53^ and the presence of produced byproducts. The chemical shifts of α, β and δ protons of residues 51, 52 and 53 for peptides 1 to 7, which are relevant for the position of representative cross-peaks in TOCSY, are given in Table S1.

In the expanded TOCSY spectrum of (KG)_5_MOG_35–55_ (Pep 5), two sets of signals were observed for residues 51, 52 and 53 (Figure 2A), indicating a modification in this peptide region. Only one set of signals was observed for the residues remote from position 53. In the spectra of (KG)_5_MOG_35–55_(Asp^53^) (Pep 6) and (KG)_5_MOG_35–55_(isoAsp^53^) (Pep 7), only one set of signals appeared for all residues (Figure 2B,C), indicating that these two peptides were not prone to any modification process. The comparison of cross-peak patterns in expanded TOCSY spectra of Pep 5, Pep 6, and Pep7 clearly demonstrate the type of modification at Asn^53^. Namely, the positions of α-β cross-peaks of Asn^53^/Asp^53^ and Tyr^51^ and α–δ cross-peaks of Arg^52^ considerably differed between Pep 6 and Pep 7 (Figure 2B,C). The positions of the additional weaker set of signals observed in expanded TOCSY region of Pep 5 (Figure 2A) were the same as the positions of the α-β cross-peaks of Asp^53^ and Tyr^51^, and the α-δ cross-peak of Arg^52^ of Pep 6 (Figure 2B). These observations clearly indicate a partial deamidation of Asn^53^ in Pep 5 to Asp^53^. The position of cross-peaks of the residues remote from position 53 was the same for all three peptides Pep 5, Pep 6, and Pep 7.

In the TOCSY region of the byproduct isolated via semi-preparative HPLC from (KG)_5_MOG_35–55_ (Pep 5) in bicarbonate buffer pH 9.0, two sets of signals appeared at the same positions as observed for Pep 5 (Table S1, Figure S16). However, the intensity ratio between the two sets of signals was different. In contrast to observations in the TOCSY spectra of Pep 5 (Figure 2A), the signals in the TOCSY spectra of byproducts corresponding to (KG)_5_MOG_35–55_(Asp^53^) (Pep 6) were stronger, and the signals corresponding to Pep 5 were weaker. That means that the byproduct of Pep 5 is Pep 6, while the presence of weaker signals of Pep 5 in the spectra of byproduct was the consequence of the not totally successful separation of byproduct from Pep 5. Namely, a small amount of Pep 5 was co-eluted with its byproduct (Pep 6).

Additional confirmation for the partial deamidation of Asn^53^ was obtained by the comparison of cross-peak patterns in the corresponding expanded TOCSY spectra of the shorter model peptides MOG_41–55_, containing Asn (Pep 1), Ala (Pep2), Asp (Pep 3), and isoAsp (Pep 4) at position 53, as recorded in bicarbonate buffer at pH 9.0 (Figure S17). Similarly to (KG)_5_MOG_35–55_, two sets of signals were observed only for residues 51, 52, and 53 of MOG_41–55_ (Pep 1) containing Asn at position 53 (Figure S17A). Only one set of signals was present in the spectra of Pep 2, Pep 3, and Pep 4 (Figure S17D, B and C, respectively). Moreover, the positions of the additional weaker set of cross-peaks of Pep 1 (Figure S17A) were the same as the positions of the α-β cross-peaks of Asp^53^ and Tyr^51^ and the α-δ cross peak of Arg^52^ of Pep 3 (Figure S17B), indicating partial deamidation of Asn^53^ to Asp^53^ in Pep 1.

Comparison of expanded TOCSY spectra of peptides 1 to 7 recorded in bicarbonate buffer pH 9.0, which are presented in Figure 2 and Figure S17, clearly demonstrated a modification of Pep 1 and Pep 5 due to partial deamidation of the Asn^53^ to Asp^53^. The peptides having Asp, isoAsp, or Ala at position 53 were stable.

### 2.4. In Vivo Evaluation of Con-Pep 8 and Con-Pep 9 Conjugated to Mannan

Con-Pep 8 conjugated with the oxidized form of mannan strongly protected C57BL/6 mice against MOG-induced Experimental Autoimmune Encephalomyelitis (EAE) in prophylactic and therapeutic protocols [31]. Moreover, Con-Pep 8 strongly protected transgenic mice expressing the human *major histocompatibility complex class II* (MHC II) MS candidate susceptibility genes DRA*0101 and DRB1*1501 and lacking all murine MHC II molecules (DR2b.Ab° mice) against MOG-EAE with prophylactic or therapeutic applications and therapeutic administration of Con-Pep 8 in mice with ongoing MOG-EAE reduced spinal cord EAE lesions, inflammatory infiltrates, microglial activation, demyelination, and neuronal damage in DR2b.Ab° mice [47].

Administration of Con-Pep 8 [OM-(KG)_5_MOG_35–55_ (Ser^42^)] or Con-Pep 9 [OM-(KG)_5_MOG_35–55_ (Ser^42^, Asp^53^)], conjugated to mannan in its oxidized form, in a prophylactic (vaccination) protocol both protected mice against the clinical and neuropathological symptoms of MOG-EAE.

**Figure 3:**
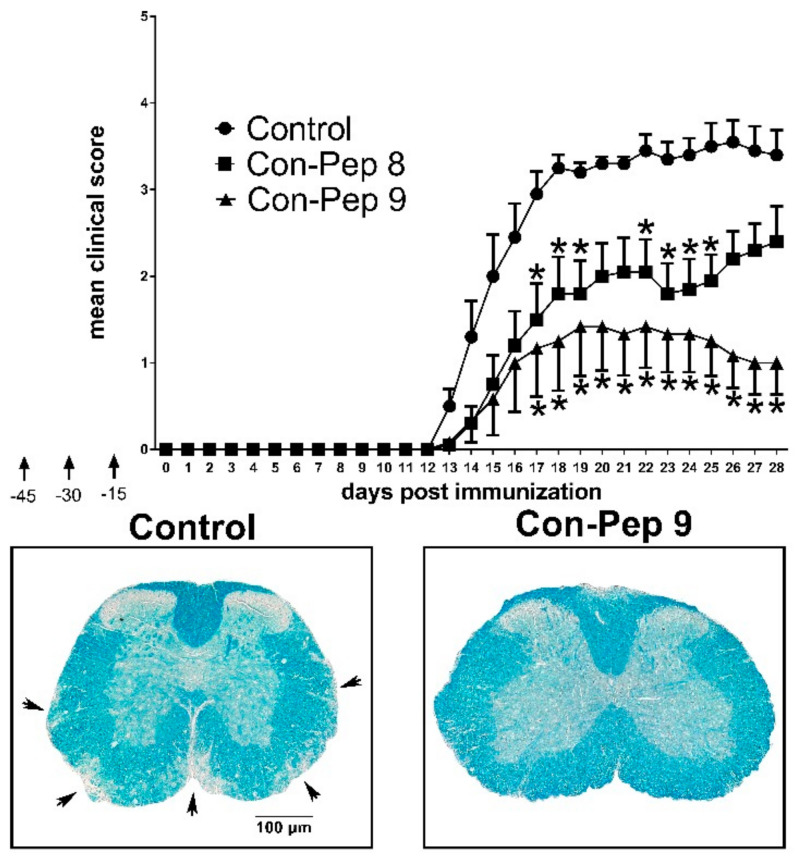
Administration of Con-Pep 8 and Con-Pep 9, conjugated to mannan in its oxidized form, in a prophylactic (vaccination) protocol attenuates the development of MOG-induced experimental autoimmune encephalomyelitis (EAE) in C57BL/6 mice. Top: Mean clinical scores of MOG-EAE in groups of mice vaccinated intradermally (i.d.) with dilute soluble Con-Pep 8, Con-Pep 9 or carbonate buffer pH 9.0 (disease control) at the indicated time points (arrows), prior to the induction of EAE by immunization with MOG/CFA/PTx (*n* = 10 for Con-Pep 8 and control groups, *n* = 6 for Con-Pep 9 group). Bottom: MOG-EAE vehicle control mice induces hallmark demyelinating lesions, as seen by Luxol Fast blue staining of spinal cord sections taken from mice sacrificed on day 28 post-immunization for EAE (left panel, arrows). Vaccination with either Con-Pep 8 (data not shown) or Con-Pep 9 prevented the development of spinal cord demyelination (right panel). Representative sections from three animals per group are shown. Statistical significance after comparisons between Con-Pep 8 and 9 treated with control groups of mice by 2-way ANOVA followed by Bonferroni post-hoc tests is shown (* *p* ≤ 0.05).

To investigate the effect of Pep 9 on the development of MOG-EAE, we delivered Con-Pep 9 or, as a positive control, Con-Pep 8 to C57BL/6 mice in a prophylactic vaccine protocol prior to the induction of EAE by immunization with MOG emulsified in CFA and administration of *Bordetella pertussis* toxin (MOG/CFA/PTx). As described previously, three intradermal (i.d.) injections of Con-Pep 8 in dilute soluble form and spaced at 15-day intervals prior to EAE induction protected mice against the clinical symptoms of EAE [31]. We show here that vaccination with Con-Pep 9 also strongly protected mice against the development of clinical EAE, to an equal extent as Pep 8, compared to vehicle-treated controls (Figure 3, top).

To determine whether amelioration of the clinical symptoms of MOG-EAE by Con-Pep 9 was associated with less severe neuropathology, mice from the different groups were sacrificed 28 days after EAE induction for neuropathological analysis of spinal cord tissues. Vehicle-treated control mice showed extensive demyelination of the spinal cord white matter characteristic of this model (Figure 3, bottom left panel). As described previously [31], vaccination of mice with Con-Pep 8 protected them against the neuropathological changes of MOG-EAE (data not shown). Here we show that vaccination of mice with Con-Pep 9 also protected against the development of neuropathological changes, with Con-Pep 9-vaccinated mice showing little or no demyelination in the spinal cord (Figure 3, bottom right panel). 

These results show that the administration of Con-Pep 9 as a prophylactic vaccine in mice inhibited the clinical and neuropathological features of MOG-EAE to an equal extent as Con-Pep 8.

## 3. Materials and Methods 

### 3.1. Synthesis

All commercially available solvents and reagents were HPLC grade, purchased from Merck (Darmstadt, Germany), Sigma-Aldrich (Schnelldorf, Germany), Fluka (Buchs, Switzerland), and Across (Geel, Belgium), and they were used without further purification. HPLC grade water was obtained from distilled water passed through a Milli-Q water purification system (Millipore Ltd., Bedford, MA, USA). CLTR-Cl resin (1% DVB, 200–400 mesh), Fmoc-protected-amino acids and piperidine were purchased from Chemical and Biopharmaceutical Laboratories of Patras, Patras, Greece. The stated resin loading capacity was determined spectrophotometrically using a UV/Visible Spectrophotometer Varian Cary 4000. ESI-MS spectra were acquired using a Waters Micromass ZQ Electrospray Platform coupled to a MassLynx 4.1 data acquisition system (Manchester, UK). HPLC chromatograms were acquired using a 1260 Infinity, Quaternary Pump VL Agilent, (Waldbronn, Germany). The ESI-MS analysis was performed using the ion spray source in positive ion mode with the following settings: (i) capillary voltage: 3.5 kV; (ii) cone voltage: 30 V; (iii) source temperature: 80oC; (iv) desolvation temperature: 300oC; (v) desolvation gas: 300 L/h; (vi) cone gas: 50 L/h.

#### Peptide Synthesis

The studied peptides (Table 1) were successfully synthesized using the solid-phase peptide synthesis (SPPS) and Fmoc/tBu methodology on CLTR-Cl resin [39,40,41,42,43,44,45,46]. The first *N^a^-*Fmoc protected amino acid, Fmoc-Lys(Boc)-OH, was esterified to the resin in the presence of DIPEA (4.5 equivalent) in DCM for 1.5 h at room temperature (RT). The remaining active site of the resin was capped using a mixture of dichloromethane (DCM) / Methanol (MeOH) / DIPEA (85/10/5; *v*/*v*/*v*) at RT. The resin was washed with *N, N-*dimethylformamide (DMF, three times × 5 mL) and isopropanol (i-PrOH; three times × 5 mL) and dried overnight under vacuum. For each peptide, 1 g CLTR-Cl resin was loaded with Fmoc-Lys(Boc)-OH (1.5 mmol), and the *N^a^*-Fmoc group was removed with 25% piperidine in DMF (*v/v*) (Scheme 1). The second amino acid, Fmoc-Gly-OH, was added (2.5 mmol) in the presence of *N,N′-*diisopropylcarbodiimide (DIC; 2.75 mmol) and 1-hydroxybenzotriazole (HOBt; 3.75 mmol) in *N,N*-dimethylacetamide (DMAC) for 4 h, followed by Fmoc deprotection with piperidine solution; three times with 25% piperidine in DMF (*v/v*) for 5 min, 15 min and 10 min respectively The coupling and deprotection cycles were repeated for all the other remaining amino acids (Scheme 1). The coupling reaction and Fmoc deprotection were demonstrated by Kaiser test and thin-layer chromatography (TLC; eluent system: AcN/H_2_O: 5/1 or 2/1; *v/v*). Then, each protected peptide was cleaved from the resin by treatment with the cleaved solution comprised of DCM/trifluoroethanol (TFE) (7/3) for 1.5 h at RT, to afford the crude side chain protected peptides. The final deprotection of the peptide was accomplished using a TFA/DCM/Anisole/Ethanodithiol (EDT) (94/4/1/1; *v/v/v/v*) solution for 5 h at RT.

The purification of each peptide was carried out on a semi-preparative reverse-phase high-performance liquid chromatography (RP-HPLC) using a Nucleosil RP-C18 column (7 μm, 250 × 4 mm, Macherey-Nagel, Duren, Germany). The separation was achieved by gradient elution from 10% AcN to 60% AcN over 45 min at a flow rate of 3 mL/min. The HPLC solvents for the analytical and semi-preparative HPLC analysis were: H_2_O with 0.08% TFA, AcN with 0.08% TFA. The final products were identified using ESI-MS and NMR spectroscopy. The purity of the synthesized peptides was determined by analytical HPLC using a Purospher RP-18 column (5μm, Hibar 100 × 4.6 mm, Darmstadt, Germany) followed by UV detection at 214.4 nm. Separation was achieved by gradient elution from 18% AcN to 40% AcN over 30 min at flow rate of 1 mL/min.

The Pep 8 and Pep 9 peptides, based on the rat/mouse sequence of MOG, were conjugated with the oxidized form of mannan (poly-mannose) to Con-Pep 8 [OM-(KG)_5_MOG_35–55_ (Ser^42^)] and Con-Pep 9 [OM-(KG)_5_MOG_35–55_ (Ser^42^, Asp^53^)] respectively as previously described [31,33,34], and they were used in the EAE animal model of MS, for further in vivo evaluation.

### 3.2. NMR Spectroscopy

The high-resolution NMR spectra were recorded on Agilent 600 MHz and Varian DirectDrive 800 MHz spectrometers (Agilent Technologies Inc., Santa Clara, CA 95051, USA) at 298 K. Cryogenic triple-resonance NMR probes were used. Ultra-precision 5 mm NMR tubes from Wilmad® were used containing peptides in a 0.7 mL aqueous solution of bicarbonate buffer (pH 9.0). The 0.1 M bicarbonate buffer solution (pH 9.0) was created using sodium bicarbonate and sodium carbonate solutions as follows: Solution A: 0.210 g Na_2_CO_3_ were dissolved in 10 mL D_2_O; Solution B: 0.168 g NaHCO_3_ were dissolved in 10 mL D_2_O. 0.8mL of solution (A) was added to 9.6ml of solution (B), and the mixture was diluted to the final volume of 50 mL with the addition of D_2_O. pH was measured with the use of a HANNA pH meter. (Calibration of pH meter was achieved with the use of calibration solutions, pH 4.0 and pH 7.0, as reported in the instruction manual).

The concentration of peptides was 1.2 mM. Water was purified with Milli-Q Water Purification System. D_2_O (“100%”) was purchased from Cambridge Isotope Laboratories. DSS-*d_6_* (0.1 mM) purchased from Sigma-Aldrich was used as an internal standard.

The proton chemical shifts of peptides 1–7 were assigned using 2D homonuclear proton experiments (Double Quantum-Filtered Correlated Spectroscopy (DQF-COSY), TOCSY, Nuclear Overhauser Effect Correlation Spectroscopy (NOESY), and Rotating-Frame Nuclear Overhauser Effect Correlation Spectroscopy (ROESY)) in combination with the 2D ^1^H–^13^C Heteronuclear Single Quantum Coherence (HSQC) experiment.

The 2D homonuclear proton spectra were acquired on a Varian DirectDrive 800 MHz spectrometer with a spectral width of 8478 Hz, 4096 data points in t_2_, 4-32 scans, 512 complex points in t_1,_ and a relaxation delay of 1.5 s. The mixing time in TOSCY was 60 ms. The mixing times in NOESY and ROESY were 150 ms and 300 ms.

The ^1^H–^13^C HSQC was recorded on an Agilent 600 MHz spectrometer with ^1^H spectral width of 6345 Hz, ^13^C spectral width of 9498 Hz, 1024 data points in t_2_, 160 scans, 128 complex points in t_1_, and a relaxation delay of 1.5 s.

All data were collected in phase-sensitive mode using pulse sequences and phase-cycling routines provided in Varian/Agilent libraries of pulse programs. In the DQF-COSY and ^1^H-^13^C HSQC experiments, gradients were used for the coherence selection. The WATERGATE pulse block was used for water suppression in DQF-COSY. The residual water signal in TOCSY, NOESY, and ROESY was suppressed using excitation sculpting, and adiabatic pulses were applied for suppression of zero quantum artifacts during mixing time.

Spectra were processed and analyzed with the Felix 2007 software package (Felix NMR Inc., San Diego, CA, USA). The spectra were zero-filled twice and apodized with a squared sine-bell function shifted by π/2 in both dimensions. In the ^13^C dimension, a linear prediction was used.

### 3.3. EAE Evaluation

#### 3.3.1. Mice

Female C57BL/6 mice aged 24 weeks were used for the prophylactic administration of peptide conjugates. Mice were bred and maintained under specific pathogen-free conditions in the experimental animal unit of the Hellenic Pasteur Institute. All animal experiments complied with ARRIVE guidelines and were carried out in accordance with the local Ethical Committee guidelines on the use of experimental animals at the Hellenic Pasteur Institute, were approved by the national authorities and conformed to EU Directive 2010/63/EU for animal experiments. Animal experiments were performed under license number 2580/29-05-2018, entitled “Induction of experimental autoimmune encephalomyelitis (EAE) for the study of immune tolerance induced by mannan-conjugated peptides”. Issued 31-05-2018 to L.P. by the Hellenic Republic Region of Attica Agricultural Economy, Veterinary and Fisheries.

#### 3.3.2. Administration of Peptides to Mice

In a prophylactic vaccination protocol, groups of adult female C57BL/6 mice (24 week-old) were injected intradermally (i.d.) on the flanks with 100 μl carbonate buffer pH 9.0 containing Con-Pep 9 (conjugated with mannan) (*n* = 6) or Con-Pep 8 (conjugated with mannan) (*n* = 10), which has been described previously [31]; 30 μg peptide (Pep 8 or Pep 9) equivalent/injection, 700 μg mannan equivalent/injection. Three consecutive injections were performed at 15-day intervals. Control mice were injected with carbonate buffer alone (*n* = 10). Immunization for the induction of EAE was performed 15 days after the last i.d. injection.

#### 3.3.3. EAE Induction

EAE was induced in vaccinated female C57BL/6 mice by subcutaneous (s.c.) tail-base injection of 38 μg of rat MOG_35–55_ (Ser^42^) (MOG) in 100 μL saline emulsified in an equal volume of complete Freund’s adjuvant (CFA), 15 days after the third vaccination injection. CFA was supplemented with 400 μg/injection of H37Ra *Mycobacterium tuberculosis* (Difco). Mice also received intraperitoneal (i.p.) injections of 200 ng of *Bordetella pertussis toxin* (PTx) (Sigma-Aldrich) at the time of immunization and 48 h later. Mice were monitored daily for the clinical signs of EAE according to the following scores: zero, normal; one, limp tail; two, hind limb weakness; three, hind limb paralysis; four, forelimb paralysis; five, moribund or dead (0.5 graduations represent intermediate scores). Mice with a score above four were euthanized and given a clinical score five for the remaining days of the experiment. All mice were allowed free access to food and water throughout the experiment.

## 4. Conclusions

In the present study, the deamidation of 35–55 Myelin Oligodendrocyte Glycoprotein (MOG_35–55_) epitope that contains the -Asn^53^-Gly^54^- sequence was investigated via HPLC, NMR, and biological studies. The 35–55 epitope of rat MOG protein induces chronic EAE in mice of the C57BL/6 strain and is widely used as an animal model for MS for in vivo biological evaluation and immunological studies of novel therapeutics [48]. Moreover, the immunogenic (KG)_5_MOG_35–55_ epitope, including the (KG)_5_ bridge in the *N*-terminal, conjugated to polysaccharide (mannan) via Schiff base, protected mice against EAE in prophylactic and therapeutic protocols [31,47]. Thus, the synthesized Pep 5 conjugated to mannan represents a potent and promising potential new therapeutic approach for the treatment of MS [31,34,36,47]. In addition to the investigation of correspondence, the unmodified form of human (KG)_5_MOG_35–55_ peptide, model peptides were synthesized based on the MOG_41–55_ epitope with modifications at position 53 (Pep 2–4) and studied to determine whether the deamidation reaction happens and to identify deaminated products. Furthermore, it was crucial to investigate the potency of these products and demonstrate how their presence affects the efficiency.

**Scheme 2:**
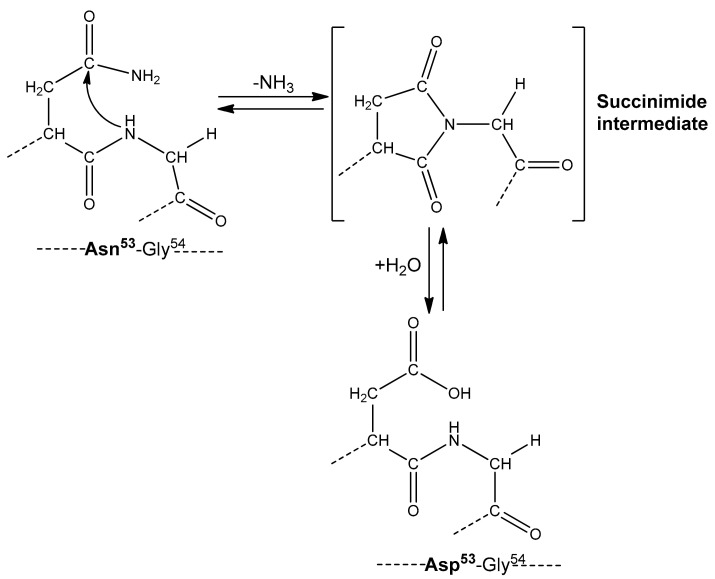
The main deamidation product of -Asn^53^-Gly^54^- sequence in MOG peptides under basic conditions.

Deamidation may occur via several pathways, as well as peptide dilution in basic conditions. The conjugation reaction between (KG)_5_MOG_35–55_ peptide with mannan occurs in basic condition (pH 9.0); thus, the deamidation reaction is an unavoidable consequence (Scheme 2). Therefore, the investigation of the immunological properties of the chemically modified peptides and the demonstration of biological action of the modified form of peptide is of great importance, since the deamidation process is associated with the maintenance of action. 

Our HPLC findings showed the presence of a new product when the peptide (with the -Asn^53^-Gly^54^- sequence) was dissolved in bicarbonate buffer solution, pH 9.0. Twenty-four hours after the dilution, the percentage of formation was almost stabilized and remained about 16% of the total. The estimated deamidation degree remained constant after three days when the analysis was repeated. The revealed new products were isolated from the mixture via HPLC and analyzed by NMR. The main NMR observations clearly indicated a partial deamidation of Asn^53^ in (KG)_5_MOG_35–55_ peptide (Pep 5) to Asp^53^. The effect of chemically modified peptides conjugated to the oxidized form of mannan (Con-Pep 8 and Con-Pep 9) was evaluated in vivo in C57BL/6 mice using the EAE animal model of MS. The deaminated derivative containing Asp at position 53 showed similar immunological activity with the native peptide. This evidence suggests that the deaminated product did not affect the biological action of the native peptide and its efficacy while tested in the animal model for the treatment of MS.

## Figures and Tables

**Table 1:** Sequence of studied peptides.

Peptide	Peptide Sequence	Abbreviation
**Pep 1**	H-Arg^41^-Pro-Pro-Phe-Ser-Arg-Val-Val-His-Leu-Tyr-Arg-Asn^53^-Gly-Lys^55^-OH	MOG_41–55_
**Pep 2**	H-Arg^41^-Pro-Pro-Phe-Ser-Arg-Val-Val-His-Leu-Tyr-Arg-**Ala^53^**-Gly-Lys^55^-OH	MOG_41–55_(Ala^53^)
**Pep 3**	H-Arg^41^-Pro-Pro-Phe-Ser-Arg-Val-Val-His-Leu-Tyr-Arg-**Asp^53^**-Gly-Lys^55^-OH	MOG_41–55_(Asp^53^)
**Pep 4**	H-Arg^41^-Pro-Pro-Phe-Ser-Arg-Val-Val-His-Leu-Tyr-Arg-**isoAsp^53^**-Gly-Lys^55^-OH	MOG_41–55_(isoAsp^53^)
**Pep 5**	H-Lys-Gly-Lys-Gly-Lys-Gly-Lys-Gly-Lys-Gly-Met^35^-Glu-Val-Gly-Trp-Tyr-Arg-Pro-Pro-Phe-Ser-Arg-Val-Val-His-Leu-Tyr-Arg-**Asn^53^**-Gly-Lys^55^-OH	(KG)_5_MOG_35–55_
**Pep 6**	H-Lys-Gly-Lys-Gly-Lys-Gly-Lys-Gly-Lys-Gly-Met^35^-Glu-Val-Gly-Trp-Tyr-Arg-Pro-Pro-Phe-Ser-Arg-Val-Val-His-Leu-Tyr-Arg-**Asp^53^**-Gly-Lys^55^-OH	(KG)_5_MOG_35–55_(Asp^53^)
**Pep 7**	H-Lys-Gly-Lys-Gly-Lys-Gly-Lys-Gly-Lys-Gly-Met^35^-Glu-Val-Gly-Trp-Tyr-Arg-Pro-Pro-Phe-Ser-Arg-Val-Val-His-Leu-Tyr-Arg-**isoAsp^53^**-Gly-Lys^55^-OH	(KG)_5_MOG_35–55_(isoAsp^53^)
**Pep 8**	H-Lys-Gly-Lys-Gly-Lys-Gly-Lys-Gly-Lys-Gly-Met^35^-Glu-Val-Gly-Trp-Tyr-Arg-**Ser^42^**-Pro-Phe-Ser-Arg-Val-Val-His-Leu-Tyr-Arg-**Asn^53^**-Gly-Lys^55^-OH	(KG)_5_MOG_35–55_ (Ser^42^)
**Pep 9**	H-Lys-Gly-Lys-Gly-Lys-Gly-Lys-Gly-Lys-Gly-Met^35^-Glu-Val-Gly-Trp-Tyr-Arg-**Ser^42^**-Pro-Phe-Ser-Arg-Val-Val-His-Leu-Tyr-Arg-**Asp^53^**-Gly-Lys^55^-OH	(KG)_5_MOG_35–55_ (Ser^42^, Asp^53^)
**Con-Pep 8**	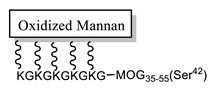	OM-(KG)_5_MOG_35–55_ (Ser^42^)
**Con-Pep 9**	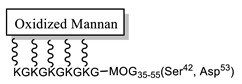	OM-(KG)_5_MOG_35–55_ (Ser^42^, Asp^53^)

OG = myelin oligodendrocyte glycoprotein, KG = Lys-Gly, OM = oxidized mannan.

## Supplementary Materials

Supplementary materials can be found at https://www.mdpi.com/1422-0067/21/20/7566/s1. Figure S1: RP-HPLC chromatogram of Pep 1 (MOG_41–55_) at 214.4 nm, at time zero and 48 h after dilution; (A) dissolved in water, (B) dissolved in bicarbonate buffer pH 9.0; t_R (main product)_: 7.47 min (83.1%) and t_R (byproduct)_: 8.27 min (13.9%), Figure S2: ESI-MS spectra of Pep 1 (MOG_41–55_) dissolved in bicarbonate buffer, pH 9.0; of main product (A) and byproduct (B); MW _theoretical (main product)_: 1826.12 Da, Figure S3: RP-HPLC chromatogram of Pep 2 [MOG_41–55_ (Ala^53^)] at 214.4 nm, at time zero and 48 h after dilution; (A) dissolved in water, (B) dissolved in bicarbonate buffer pH 9.0; t_R (main product)_: 8.45 min (100%) and t_R (byproduct)_: do not observed, Figure S4: ESI-MS spectra of Pep 2 [MOG_41–55_ (Ala^53^)]; MW _theoretical (main product)_: 1783.11 Da, Figure S5: RP-HPLC chromatogram of Pep 3 [MOG_41–55_ (Asp^53^)] at 214.4 nm, at time zero and 48 h after dilution; (A) dissolved in water, (B) dissolved in bicarbonate buffer pH 9.0; t_R (main product)_: 8.3 min (100%) and t_R (byproduct)_: do not observed; (C) RP-HPLC chromatogram after co-injection of MOG_41–55_ (Pep 1) (dissolved in bicarbonate buffer) and MOG_41–55_ (Asp^53^) (Pep 3) (dissolved in water) at 214.4 nm, Figure S6: ESI-MS spectra of Pep 3 [MOG_41–55_ (Asp^53^)]; MW _theoretical (main product)_: 1827.12 Da, Figure S7: RP-HPLC chromatogram of Pep 4 [MOG_41–55_ (isoAsp^53^)] at 214.4 nm, dissolved in water; t_R (main product)_: 7.6 min (100%), Figure S8: ESI-MS spectra of Pep 4 [MOG_41–55_ (isoAsp^53^)]; MW _theoretical (main product)_: 1827.12 Da, Figure S9: RP-HPLC chromatogram of Pep 5 [(KG)_5_MOG_35–55_] at 214.4 nm, at time zero and 48 h after dilution; (A) dissolved in water, (B) dissolved in bicarbonate buffer pH 9.0; t_R (main product)_: 13.3 min (88.6%) and t_R (byproduct)_: 13.9 min (11.4%), Figure S10: ESI-MS spectra of Pep 5 [(KG)_5_MOG_35–55_] dissolved in bicarbonate buffer, pH 9.0; of main product (A) and byproduct (B). MW _theoretical (main product)_: 3518.1 Da, Figure S11: RP-HPLC chromatogram of Pep 6 [(KG)_5_MOG_35–55_(Asp^53^)] at 214.4 nm, at 48 h after dilution; (A) dissolved in water, (B) dissolved in bicarbonate buffer pH 9.0; t_R (main product)_: 13.8 min, Figure S12: RP-HPLC chromatogram of Pep 7 [(KG)_5_MOG_35–55_(isoAsp^53^)] at 214.4 nm, at 48 h after dilution; (A) dissolved in water, (B) dissolved in bicarbonate buffer pH 9.0; t_R (main product)_: 13.3 min, Figure S13: RP-HPLC chromatogram of co-injection [(KG)_5_MOG_35–55_(Asn^53^)-(KG)_5_MOG_35–55_(Asp^53^)] at 214.4 nm, at 48 h after dilution in bicarbonate buffer pH 9.0; t_R (pep 5)_: 13.3 min, t_R (pep 6)_: 13.9 min, Figure S14: RP-HPLC chromatogram of Pep 8 [(KG)_5_MOG_35–55_(Ser^42^)] at 214.4 nm, at time zero after dilution; (A) dissolved in water, (B) dissolved in bicarbonate buffer pH 9.0, Figure S15: ESI-MS spectra of Pep 8 [(KG)_5_MOG_35–55_(Ser^42^)] dissolved in bicarbonate buffer, pH 9.0; of main product (A) and byproduct (B); MW _theoretical (main product)_: 3508.07 Da; MW _theoretical (by product)_: 3509.06 Da, Figure S16: Expanded regions of TOCSY spectra of: (A) (KG)_5_MOG_35–55_ (Pep 5) and (B) isolated byproduct, after semipreparative-HPLC purification, (KG)_5_MOG_35–55_(Asp^53^) (Pep 6) of (KG)_5_MOG_35–55_ (Pep 5) recorded in bicarbonate buffer, pH 9.0, Figure S17: Expanded regions of TOCSY spectra of: (A) MOG_41–55_ (Pep 1), (B) MOG_41–55_(Asp^53^) (Pep 3), (C) MOG_41–55_(isoAsp^53^) (Pep 4) and (D) MOG_41–55_(Ala^53^) (Pep 2) recorded in bicarbonate buffer, pH 9.0, Table S1: Chemical shifts in ppm of α, β protons of residues 51 and 53 and α, δ protons of residue 52 for peptides 1 to 7 in bicarbonate buffer (pH 9.0) referenced according to DSS-*d_6_*. These chemical shifts define the position of representative cross-peaks in expanded TOCSY spectra (Figure 2, Figures S16 and S17) relevant for the explanation of degradation of peptides containing Asn at position 53.

## Abbreviations

MOG	Myelin Oligodendrocyte Glycoprotein
MS	Multiple Sclerosis
EAE	Experimental Autoimmune Encephalomyelitis
SPPS	Solid Phase Peptide Synthesis
RP-HPLC	Reverse Phase-High Performance Liquid Chromatography
ESI-MS	Electron Spray Ionization-Mass Spectrometry
DQF-COSY	Double Quantum-Filtered Correlated Spectroscopy
TOCSY	Total Correlated Spectroscopy
NOESY	Nuclear Overhauser Effect Correlation Spectroscopy
ROESY	Rotating-Frame Nuclear Overhauser Effect Correlation Spectroscopy
HSQC	Heteronuclear Single Quantum Coherence
