# Supplementary material for: Characterization of Asparagine Deamidation in Immunodominant Myelin Oligodendrocyte Glycoprotein Peptide Potential Immunotherapy for the Treatment of Multiple Sclerosis
Source: Int J Mol Sci. 2020 Oct 13;21(20):7566. doi: 10.3390/ijms21207566 (PMC7593956; doi:10.3390/ijms21207566)
Supplement: Supplementary file 1 [file ijms-21-07566-s001.pdf]

Supplementary Materials

Characterization of Asparagine Deamidation in Immunodominant Myelin Oligodendrocyte Glycoprotein Peptide Potential Immunotherapy for the Treatment of Multiple Sclerosis

RP-HPLC Conditions:

i) Temperature: 25 ^{o}C

ii) Column: Purospher RP-18 (5μm, Hibar 100-4, 6 mm),

iii) Solvents: H_2O (0.08% TFA), AcN (0.08% TFA),

iv) Gradient elution: from 18% AcN to 40% AcN over 30 min

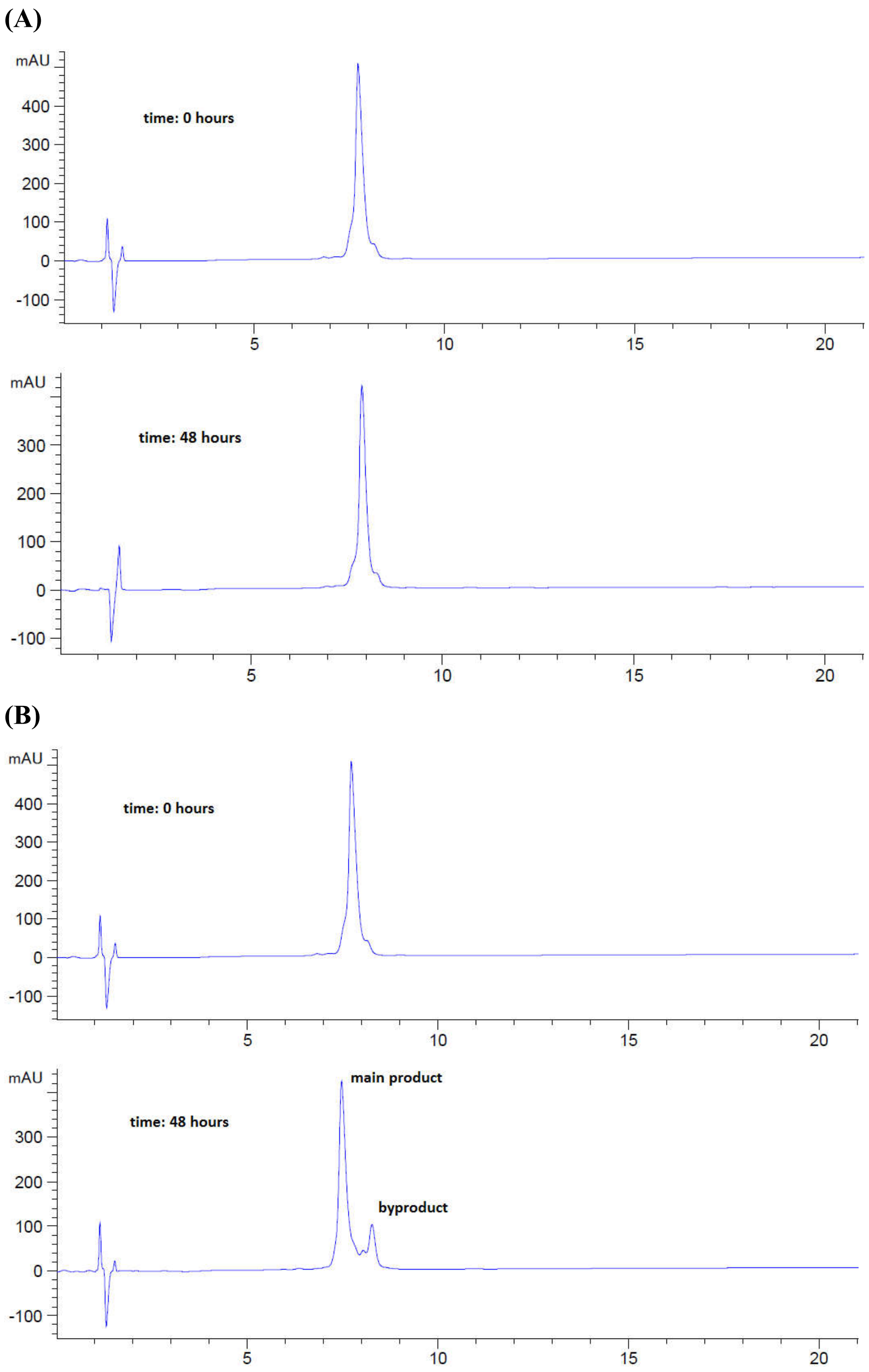

Figure S1: RP-HPLC chromatogram of Pep 1 (MOG_{4155}) at 214.4 nm, at time zero and 48 h after dilution; **(A)** dissolved in water, **(B)** dissolved in bicarbonate buffer pH 9.0

$t_{R\ (main\ product)}$: 7.47 min (83.1%) and $t_{R\ (byproduct)}$: 8.27 min (13.9%)

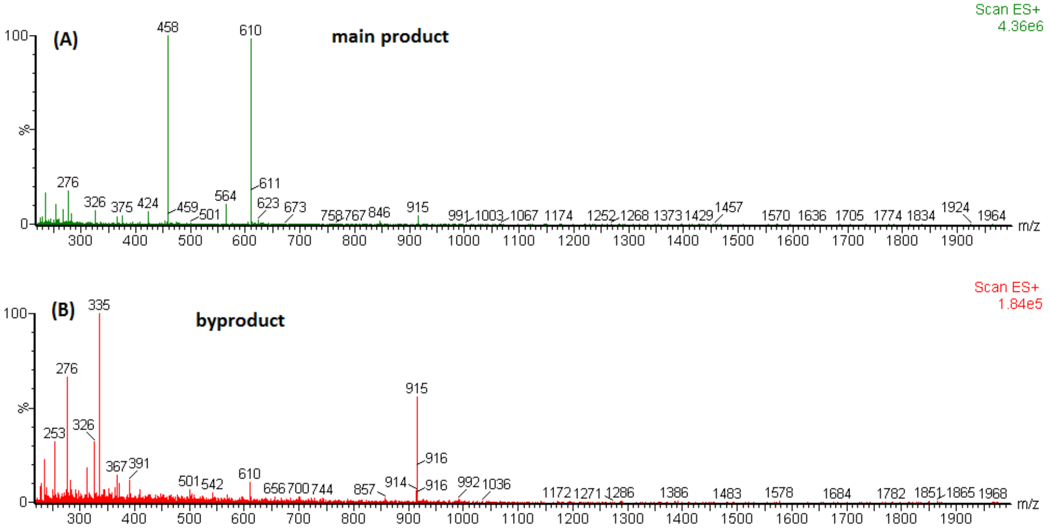

Figure S2: ESI-MS spectra of **Pep 1** (MOG$_{4155}$) dissolved in bicarbonate buffer, pH 9.0; of main product **(A)** and byproduct **(B)**

MW $_{\text{theoretical (main product)}}$: 1826.12 Da

(A)

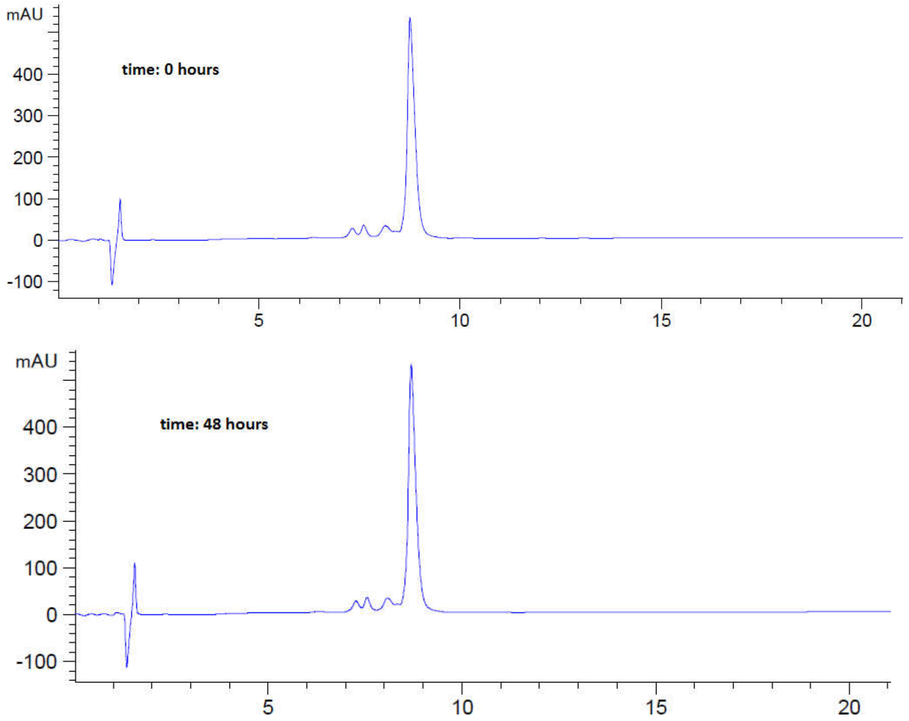

(B)

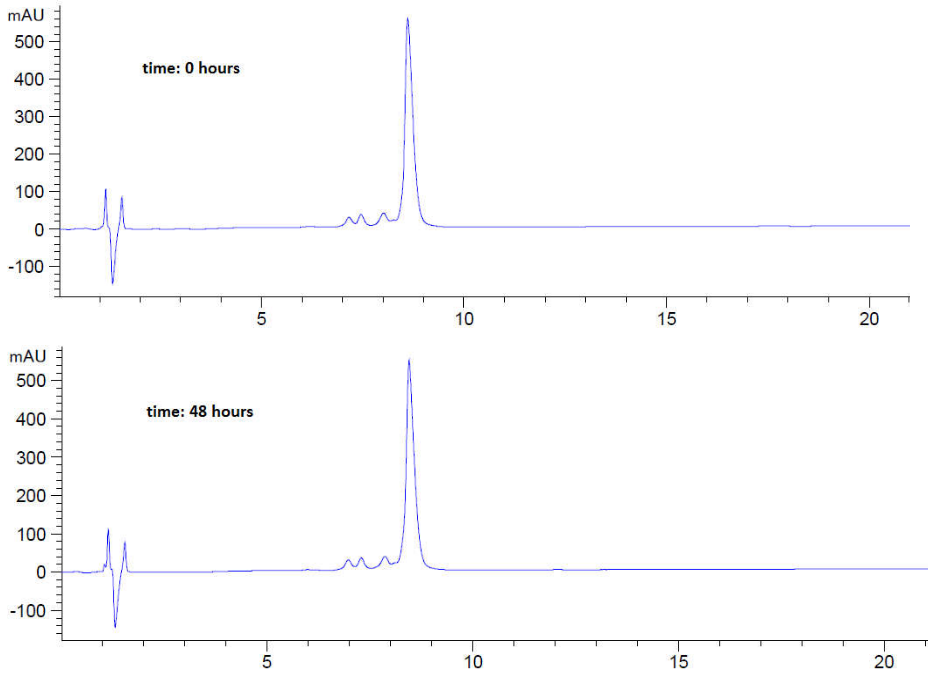

Figure S3: RP-HPLC chromatogram of **Pep 2** [MOG$_{4155}$ (Ala^{53})] at 214.4 nm, at time zero and 48 h after dilution; **(A)** dissolved in water, **(B)** dissolved in bicarbonate buffer pH 9.0

$t_{R\ (main\ product)}$: 8.45 min (100%) and $t_{R\ (byproduct)}$: do not observed

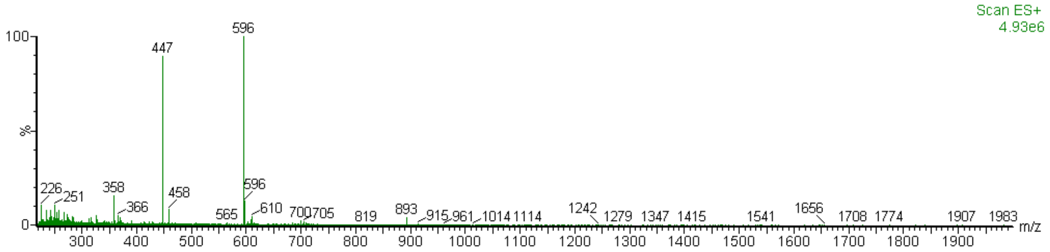

Figure S4: ESI-MS spectra of **Pep 2** [MOG_{4155} (Ala^{53})]

$MW_{\text{theoretical (main product)}}$: 1783.11 Da

(A)

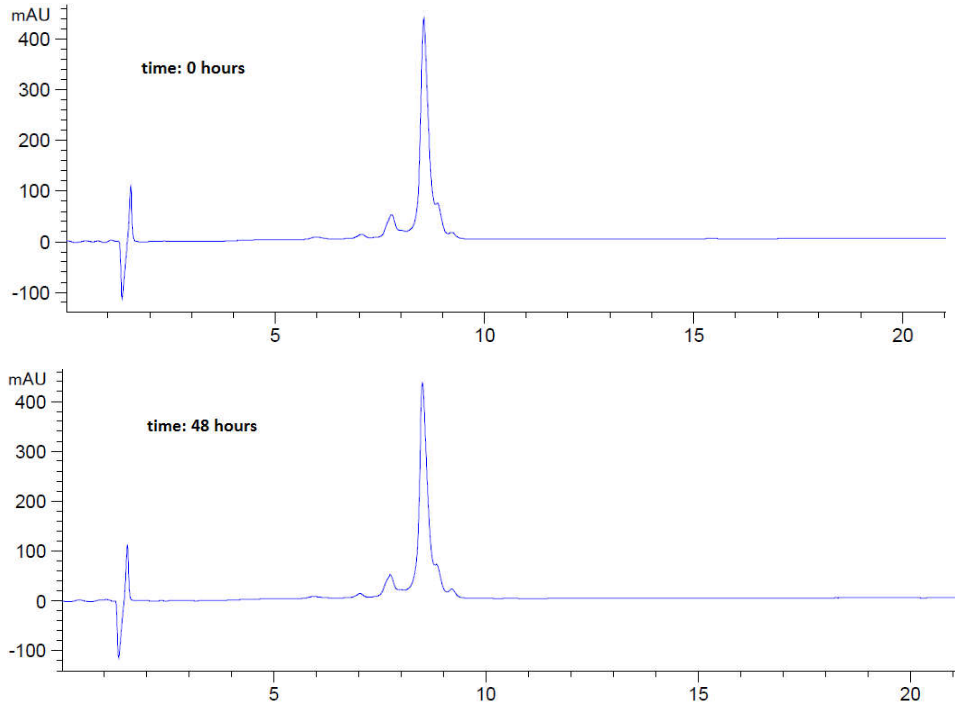
mAU
400
300
200
100
0
-100
time: 0 hours
5
10
15
20
mAU
400
300
200
100
0
-100
time: 48 hours
5
10
15
20

(B)

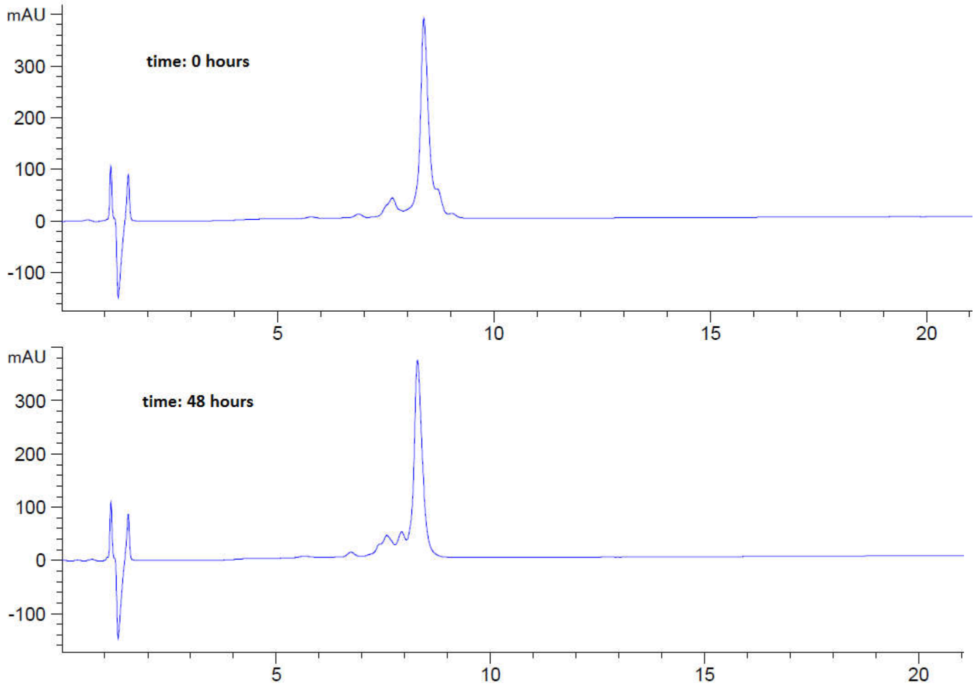
mAU
300
200
100
0
-100
time: 0 hours
5
10
15
20
mAU
300
200
100
0
-100
time: 48 hours
5
10
15
20

(C)

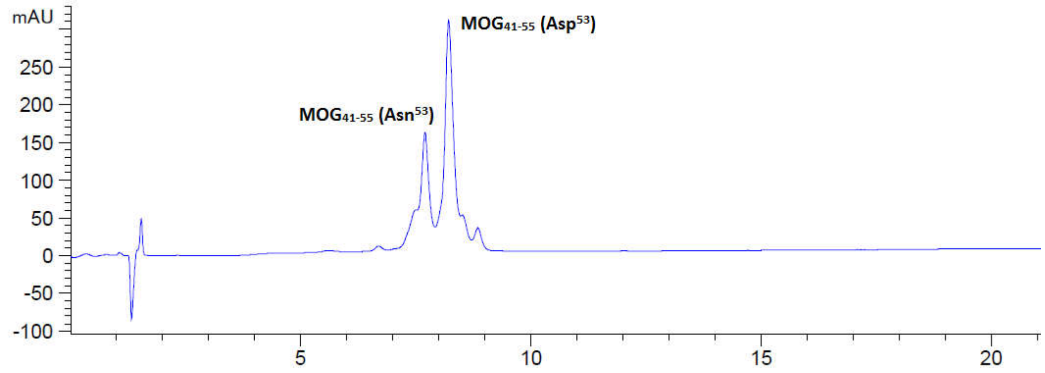

Figure S5: RP-HPLC chromatogram of **Pep 3** [MOG4155 (Asp[53])] at 214.4 nm, at time zero and 48 h after dilution; **(A)** dissolved in water, **(B)** dissolved in bicarbonate buffer pH 9.0; $t_{R\ (main\ product)}$: 8.3 min (100%) and $t_{R\ (byproduct)}$: do not observed

(C) RP-HPLC chromatogram after co-injection of MOG4155 **(Pep 1)** (dissolved in bicarbonate buffer) and MOG4155 (Asp[53]) **(Pep 3)** (dissolved in water) at 214.4 nm

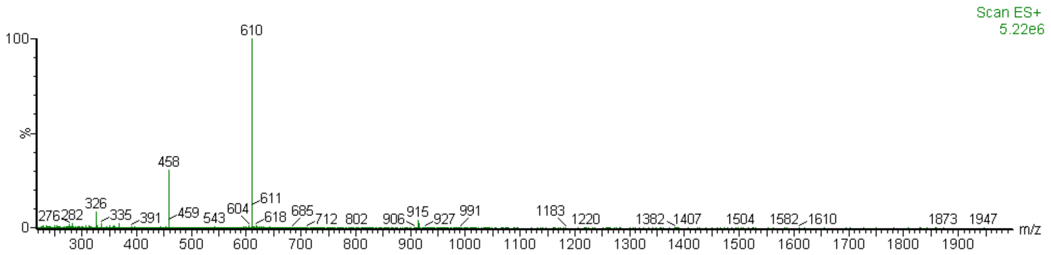

Figure S6: ESI-MS spectra of **Pep 3** [MOG4155 (Asp[53])]

$MW_{theoretical\ (main\ product)}$: 1827.12 Da

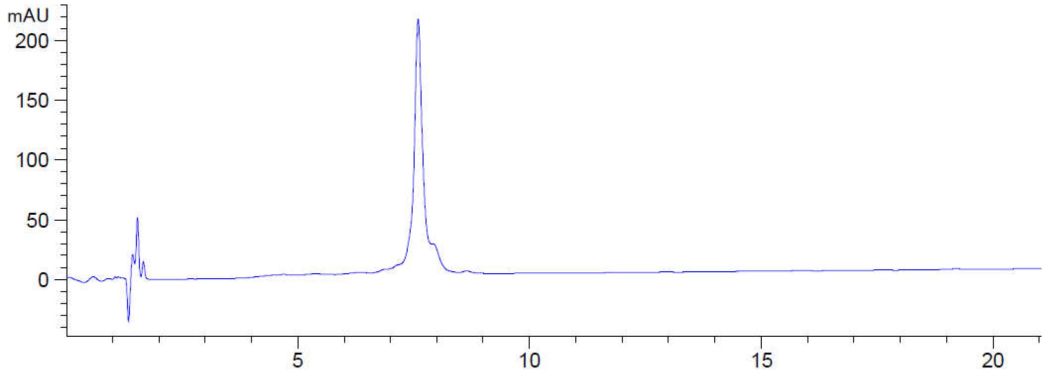

Figure S7: RP-HPLC chromatogram of **Pep 4** [MOG4155 (isoAsp[53])] at 214.4 nm, dissolved in water

$t_{R\ (main\ product)}$: 7.6 min (100%)

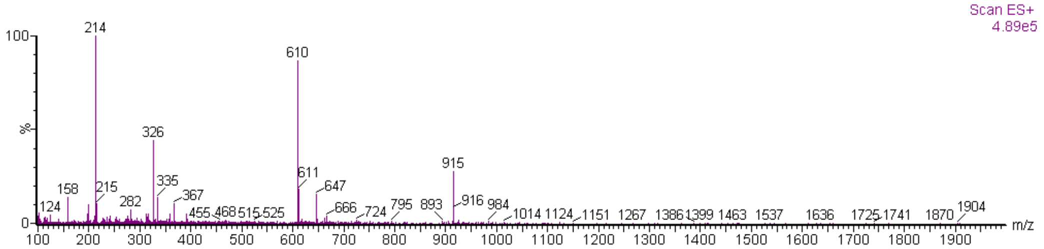

Figure S8: ESI-MS spectra of **Pep 4** [MOG$_{4155}$ (isoAsp^{53})]

MW $_{\text{theoretical (main product)}}$: 1827.12 Da

(A)

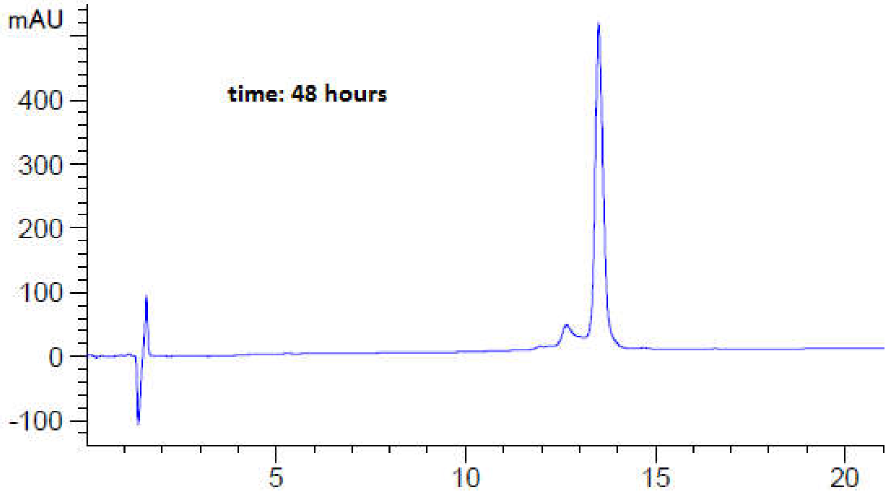

(B)

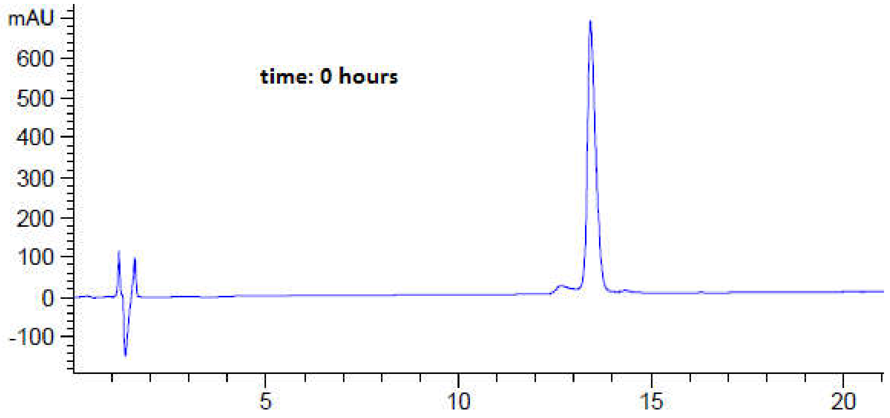

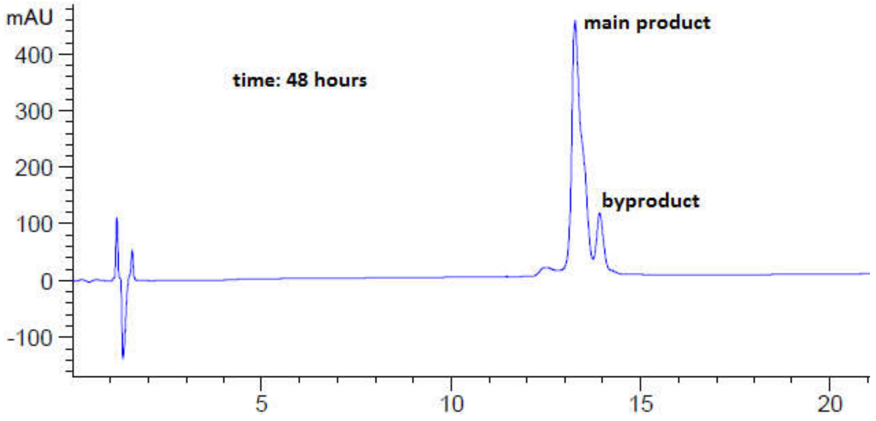

Figure S9: RP-HPLC chromatogram of **Pep 5** [(KG)$_5$MOG$_{3555}$] at 214.4 nm, at time zero and 48 h after dilution; **(A)** dissolved in water, **(B)** dissolved in bicarbonate buffer pH 9.0

$t_{R\ (main\ product)}$: 13.3 min (88.6%) and $t_{R\ (byproduct)}$: 13.9 min (11.4%)

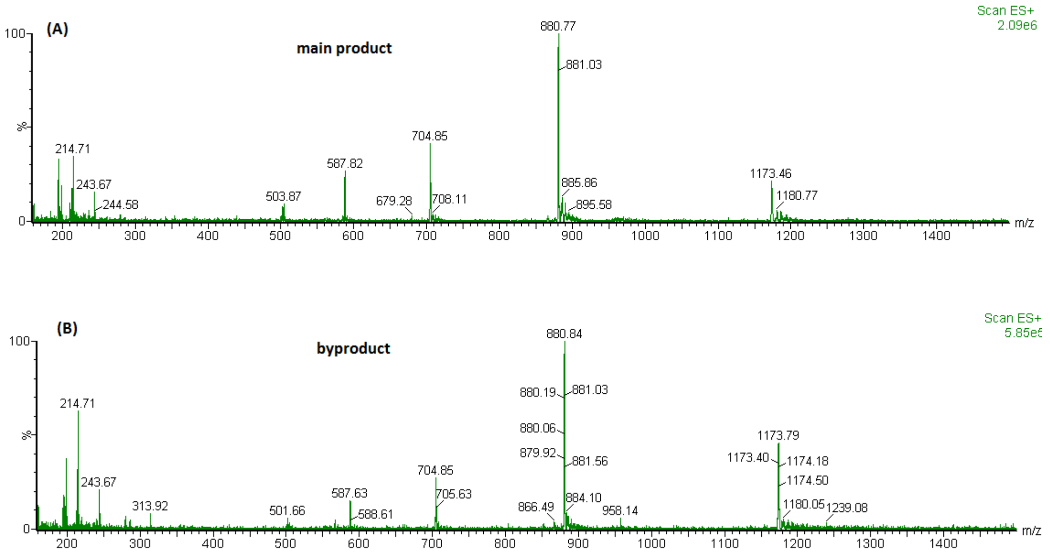

Figure S10: ESI-MS spectra of **Pep 5** [(KG)$_5$MOG$_{3555}$] dissolved in bicarbonate buffer, pH 9.0; of main product **(A)** and byproduct **(B)**

MW $_{theoretical\ (main\ product)}$: 3518.1 Da

(A)

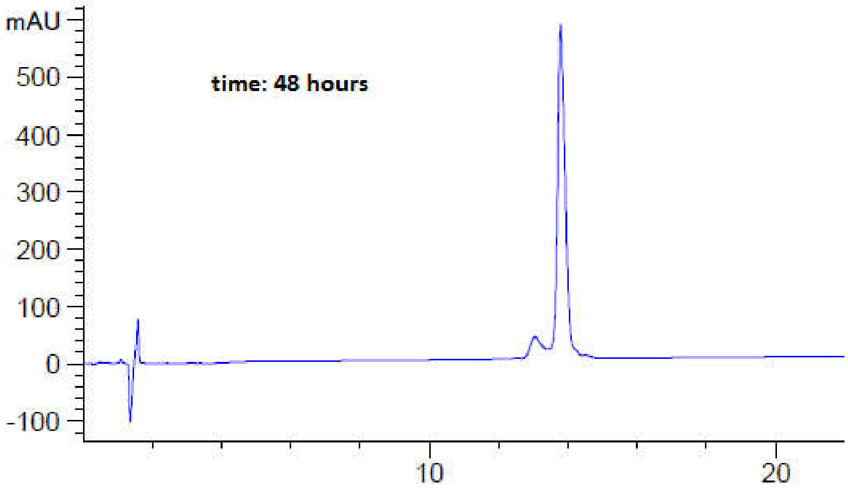

(B)

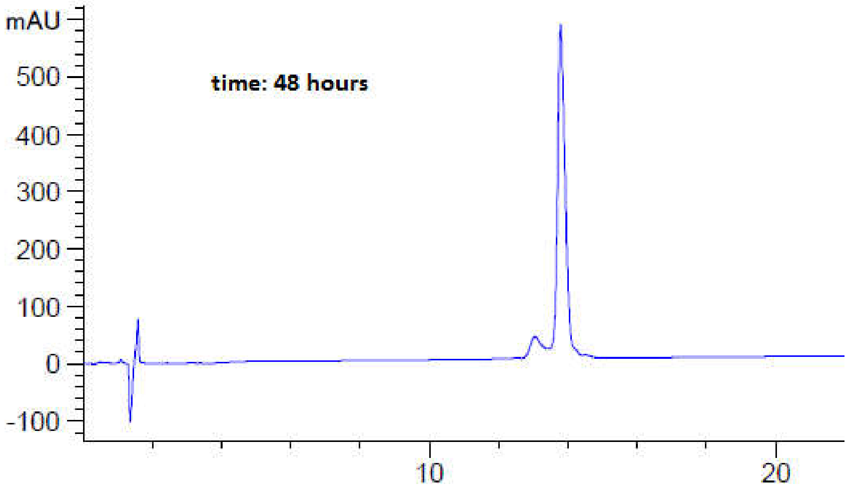

Figure S11: RP-HPLC chromatogram of **Pep 6** [(KG)$_5$MOG$_{3555}$(Asp^{53})] at 214.4 nm, at 48 h after dilution; **(A)** dissolved in water, **(B)** dissolved in bicarbonate buffer pH 9.0

$t_{R\ (main\ product)}$: 13.8 min

(A)

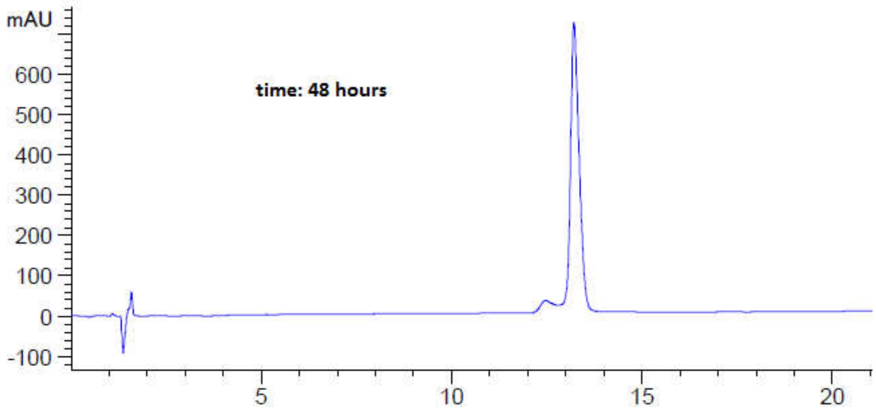

(B)

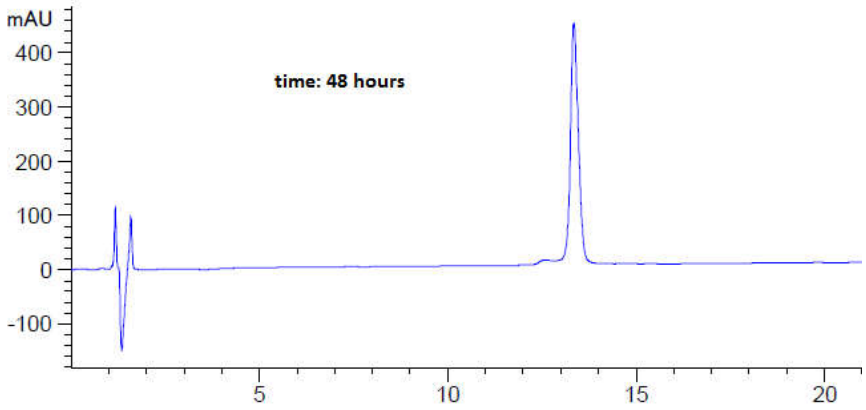

Figure S12: RP-HPLC chromatogram of **Pep 7** [$(KG)_5MOG_{3555}(isoAsp^{53})$] at 214.4nm, at 48 h after dilution; **(A)** dissolved in water, **(B)** dissolved in bicarbonate buffer pH 9.0

$t_{R\ (main\ product)}$: 13.3 min

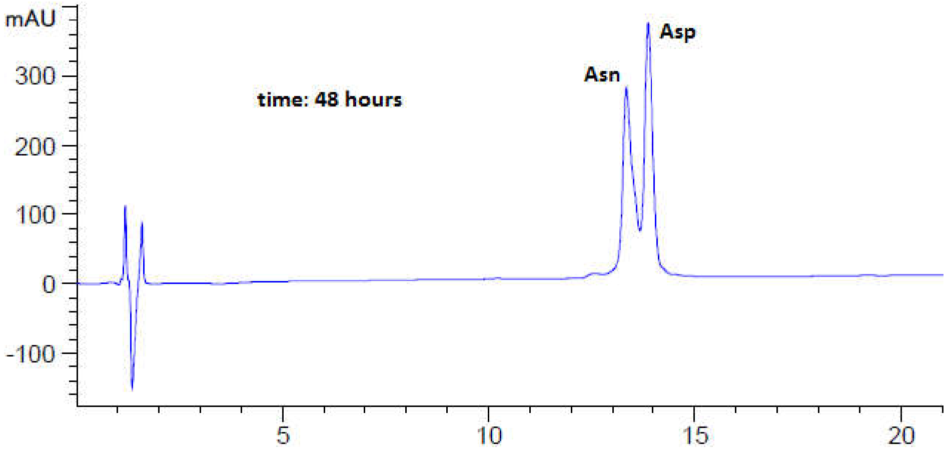

Figure S13: RP-HPLC chromatogram of co-injection [$(KG)_5MOG_{3555}(Asn^{53})$-$(KG)_5MOG_{3555}(Asp^{53})$] at 214.4 nm, at 48 h after dilution in bicarbonate buffer pH 9.0 $t_{R\ (\mathbf{pep\ 5})}$: 13.3 min, $t_{R\ (\mathbf{pep\ 6})}$: 13.9 min

(A)

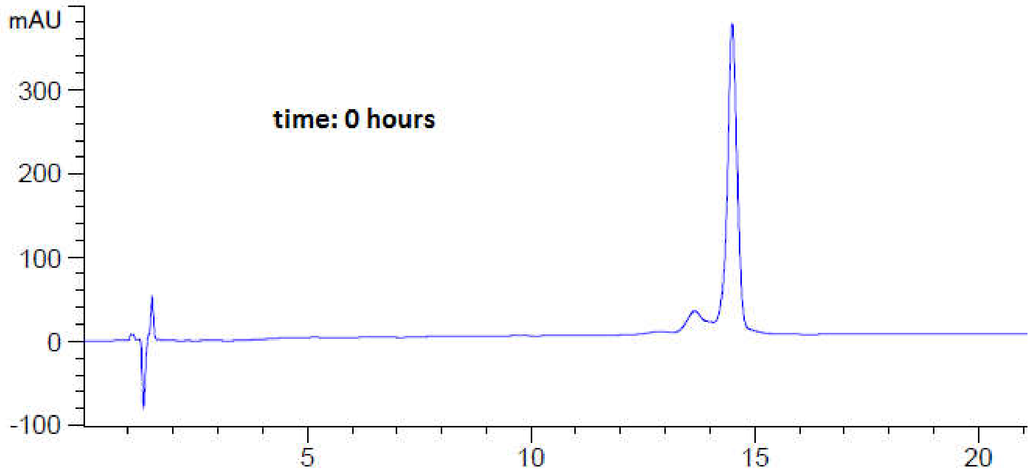

(B)

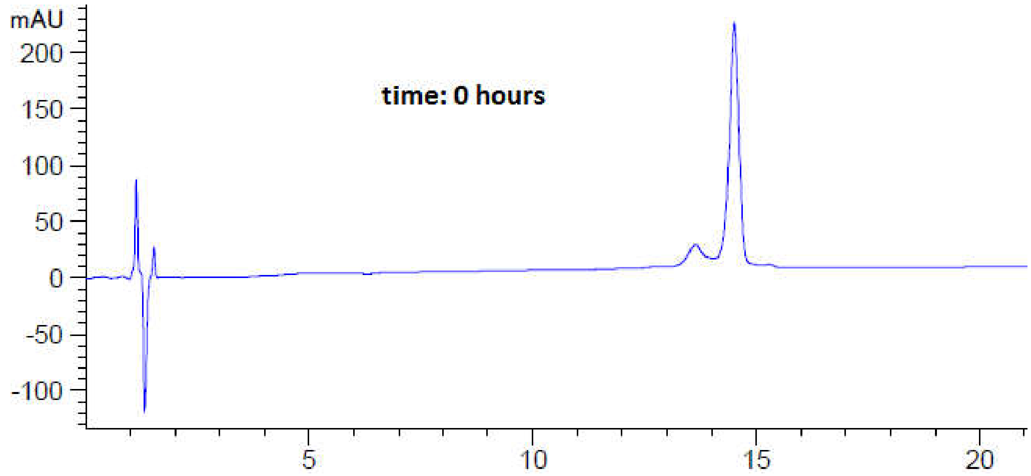

Figure S14: RP-HPLC chromatogram of Pep 8 [$(KG)_5MOG_{3555}(Ser^{42})$] at 214.4 nm, at time zero after dilution; **(A)** dissolved in water, **(B)** dissolved in bicarbonate buffer pH 9.0

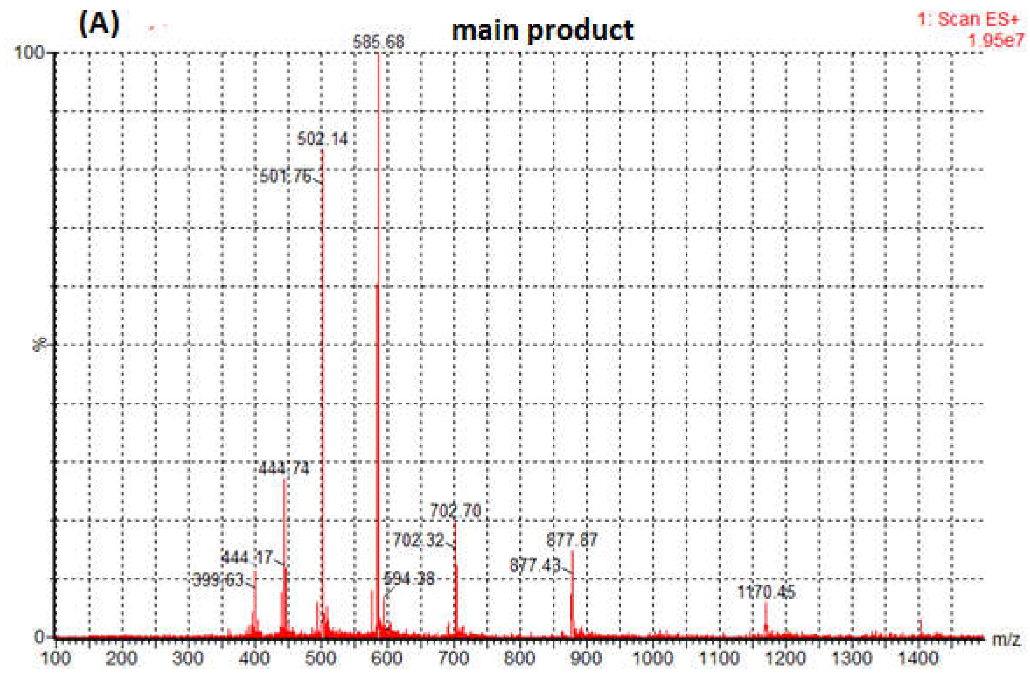

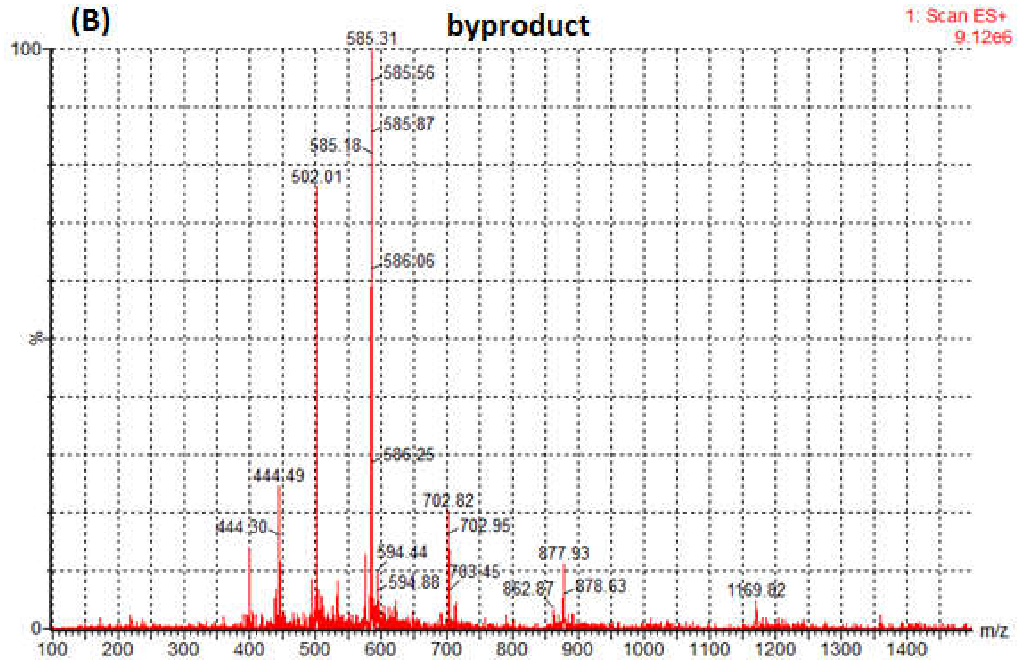

Figure S15: ESI-MS spectra of **Pep 8** [(KG)$_5$MOG$_{3555}$(Ser^{42})] dissolved in bicarbonate buffer, pH 9.0; of main product **(A)** and byproduct **(B)**

MW $_{\text{theoretical (main product)}}$: 3508.07 Da

MW $_{\text{theoretical (by product)}}$: 3509.06 Da

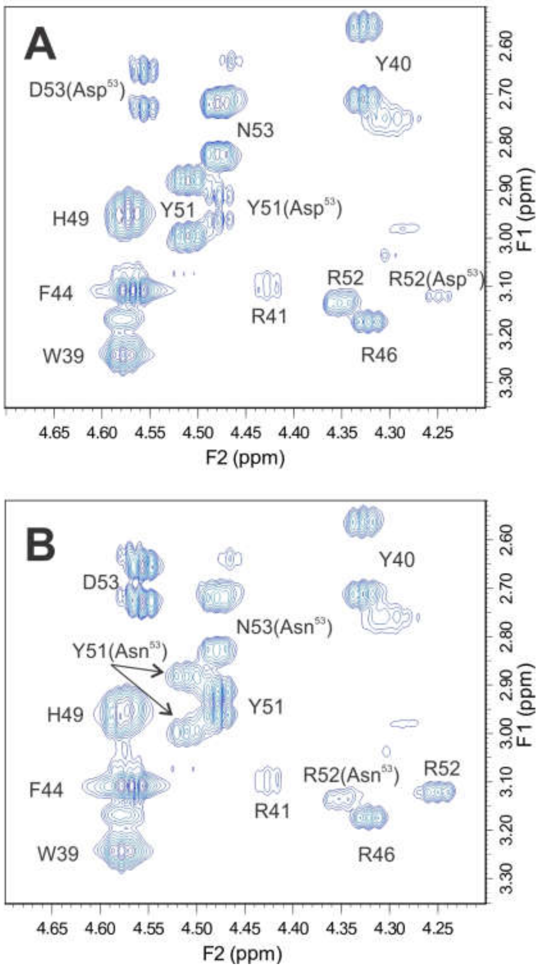

Figure S16: Expanded regions of TOCSY spectra of: **(A)** $(KG)_5MOG_{3555}$ **(Pep 5)** and **(B)** isolated byproduct, after semipreparative-HPLC purification, $(KG)_5MOG_{35-55}(Asp^{53})$ (**Pep 6**) of $(KG)_5MOG_{3555}$ **(Pep 5)** recorded in bicarbonate buffer, pH 9.0

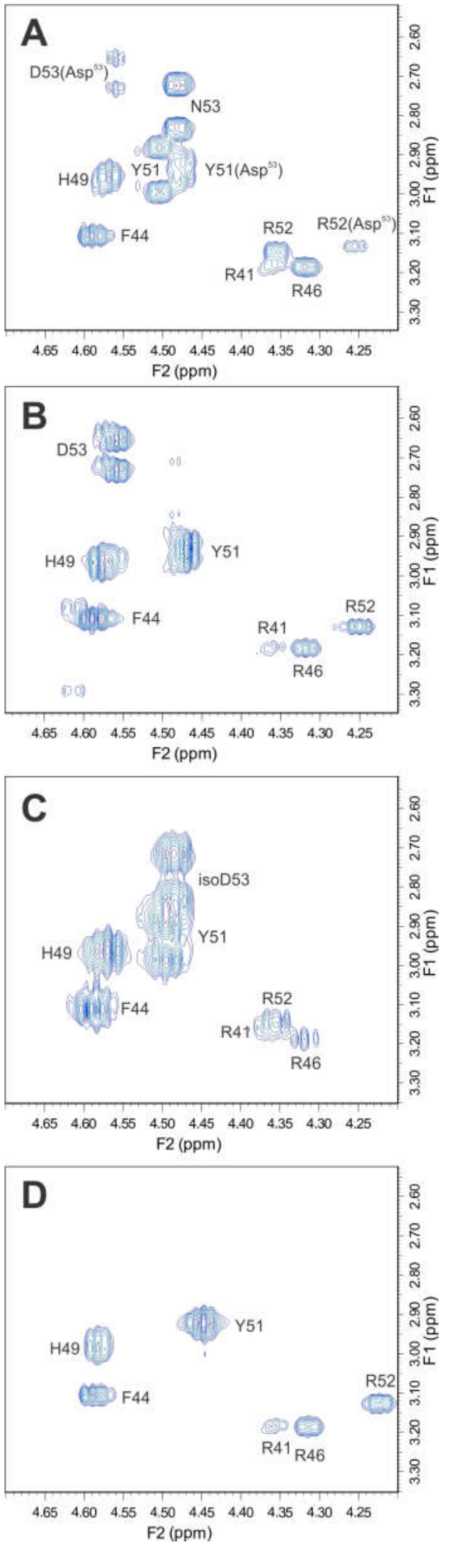

Figure S17: Expanded regions of TOCSY spectra of: **(A)** MOG$_{4155}$ **(Pep 1), (B)** MOG$_{4155}$(Asp^{53}) **(Pep 3)**, **(C)** MOG$_{4155}$(isoAsp^{53}) **(Pep 4)** and **(D)** MOG$_{4155}$(Ala^{53}) **(Pep 2)** recorded in bicarbonate buffer, pH 9.0

Table S1: Chemical shifts in ppm of α, β protons of residues 51 and 53 and α, δ protons of residue 52 for peptides 1 to 7 in bicarbonate buffer (pH 9.0) referenced according to DSS-d_6. These chemical shifts define the position of representative cross-peaks in expanded TOCSY spectra (Figures 2, S16 and S17) relevant for the explanation of degradation of peptides containing Asn at position 53

	**Pep 1** MOG_{41-55}		**Pep 2** $MOG_{41-55}(Ala^{53})$	**Pep 3** $MOG_{41-55}(Asp^{53})$	**Pep 4** $MOG_{41-55}(isoAsp^{53})$
	major	minor			
Tyr51 H^{α}	4.50	4.47	4.45	4.47	4.50
Tyr51 H^{β}	2.88 2.30	2.92 2.95	2.92	2.91 2.95	2.86 2.98
Arg52 H^{α}	4.36	4.25	4.22	4.25	4.38
Arg52 H^{δ}	3.15	3.13	3.12	3.12	3.16
Asn53 H^{α}	4.48				
Asn53 H^{β}	2.72 2.83				
Asp53 H^{α}		4.56		4.56	
Asp53 H^{β}		2.66 2.73		2.65 2.73	
isoAsp53 H^{α}					4.48
isoAsp53 H^{β}					2.72 2.84

	**Pep 5** $(KG)_5MOG_{35-55}$		**isolated byproduct of Pep 5**		**Pep 6** $(KG)_5MOG_{35-55}(Asp^{53})$	**Pep 7** $(KG)_5MOG_{35-55}(isoAsp^{53})$
	major	minor	major	minor		
Tyr51 H^{α}	4.51	4.48	4.48	4.51	4.48	4.52
Tyr51 H^{β}	2.30, 2.88	2.91 2.96	2.91 2.96	2.30 2.88	2.91 2.96	2.88 3.00
Arg52 H^{α}	4.35	4.25	4.25	4.35	4.25	4.35
Arg52 H^{δ}	3.13	3.12	3.12	3.13	3.12	3.13
Asn53 H^{α}	4.48			4.48		
Asn53 H^{β}	2.72 2.82			2.72 2.83		
Asp53 H^{α}		4.56	4.56		4.56	
Asp53 H^{β}		2.65 2.73	2.65 2.73		2.66 2.73	
isoAsp53 H^{α}						4.48
isoAsp53 H^{β}						2.72 2.82
